# Targeted transcutaneous spinal cord stimulation promotes persistent recovery of upper limb strength and tactile sensation in spinal cord injury: a pilot study

**DOI:** 10.3389/fnins.2023.1210328

**Published:** 2023-07-07

**Authors:** Santosh Chandrasekaran, Nikunj A. Bhagat, Richard Ramdeo, Sadegh Ebrahimi, Pawan D. Sharma, Doug G. Griffin, Adam Stein, Susan J. Harkema, Chad E. Bouton

**Affiliations:** ^1^Neural Bypass and Brain Computer Interface Laboratory, Institute of Bioelectronic Medicine, Feinstein Institutes for Medical Research, Northwell Health, Manhasset, NY, United States; ^2^Department of Physical Medicine and Rehabilitation, University of Texas Health Science Center, Houston, TX, United States; ^3^Kentucky Spinal Cord Injury Research Center, University of Louisville, Louisville, KY, United States; ^4^Northwell Health STARS Rehabilitation, East Meadow, NY, United States; ^5^Department of Physical Medicine and Rehabilitation, Donald and Barbara Zucker School of Medicine at Hofstra, Northwell Health, Manhasset, NY, United States; ^6^Department of Bioengineering, University of Louisville, Louisville, KY, United States; ^7^Frazier Rehabilitation Institute, University of Louisville Health, Louisville, KY, United States; ^8^Department of Neurological Surgery, University of Louisville, Louisville, KY, United States; ^9^Donald and Barbara Zucker School of Medicine at Hofstra/Northwell, Manhasset, NY, United States

**Keywords:** spinal cord injury, spinal cord stimulation, brain computer interface, selective stimulation, neuromodulation, neurostimulation devices, electrode array, paralysis

## Abstract

Long-term recovery of limb function is a significant unmet need in people with paralysis. Neuromodulation of the spinal cord through epidural stimulation, when paired with intense activity-based training, has shown promising results toward restoring volitional limb control in people with spinal cord injury. Non-invasive neuromodulation of the cervical spinal cord using transcutaneous spinal cord stimulation (tSCS) has shown similar improvements in upper-limb motor control rehabilitation. However, the motor and sensory rehabilitative effects of activating specific cervical spinal segments using tSCS have largely remained unexplored. We show in two individuals with motor-complete SCI that targeted stimulation of the cervical spinal cord resulted in up to a 1,136% increase in exerted force, with weekly activity-based training. Furthermore, this is the first study to document up to a 2-point improvement in clinical assessment of tactile sensation in SCI after receiving tSCS. Lastly, participant gains persisted after a one-month period void of stimulation, suggesting that targeted tSCS may lead to persistent recovery of motor and sensory function.

## Introduction

Spinal cord injury can result in paralysis due to the disruption in the transmission of neural signals. More than half of spinal cord injuries (SCIs) occur at the cervical level (NSCISC and University of Alabama at Birmingham, 2021), and regaining voluntary control of the hand and arm is the highest priority in such cases (Anderson, 2004). However, the chances of regaining hand and arm function are exceedingly low beyond 12–18 months post injury (Fawcett et al., 2007).

Neuromodulation of the lumbar spinal cord using epidurally placed electrodes has recently shown great promise in facilitating voluntary movements of the lower limb during stimulation after SCI, in rats (van den Brand et al., 2012), non-human primates (Capogrosso et al., 2016) and humans (Harkema et al., 2011; Angeli et al., 2014, 2018; Gill et al., 2018; Wagner et al., 2018). Additionally, when paired with intense motor training, lumbar epidural stimulation has demonstrated recovery of voluntary motor control even in the absence of stimulation (Wagner et al., 2018; Peña Pino et al., 2020). However, such benefits are generally restricted to muscles that have at least some preserved motor function (Huang et al., 2022).

Epidural stimulation has been shown to primarily engage the large-to-medium size sensory afferent fibers present in the roots and dorsal column of the spinal cord (Rattay et al., 2000). Local spinal circuits constitute of these afferent fibers forming synaptic connections with spinal interneurons and motoneurons. Pharmacological and computational experiments (Capogrosso et al., 2013) suggest that activation of the dorsal fibers increases the excitability of the local spinal circuitry including the efferent fibers. Expanding this idea further, recent studies have demonstrated that epidural stimulation targeting cervical spinal cord could activate (Greiner et al., 2021) and restore upper limb movement (Lu et al., 2016). Though extremely promising, the invasive nature of epidural stimulation presents a hurdle for clinical translation and persistent rehabilitation (James et al., 2018; Taccola et al., 2020).

Meanwhile, non-invasive stimulation of the spinal cord using transcutaneous electrodes has been demonstrated to evoke voluntary movements in both upper (Freyvert et al., 2018; Gad et al., 2018; Inanici et al., 2021) and lower limbs (Sayenko et al., 2019). Moreover, recent evidence suggests that transcutaneous spinal cord stimulation (tSCS) also exerts its neuromodulatory effect on motoneurons trans-synaptically via activation of large-to-medium size sensory afferent fibers (Ladenbauer et al., 2010; Hofstoetter et al., 2018) and can also improve descending supraspinal drive (Guiho et al., 2021). Only recently, has it been shown that pairing tSCS at the cervical levels with intense motor training can result in sustained improvements in hand and arm function (Benavides et al., 2020; Zhang et al., 2020; Inanici et al., 2021). Taken together, tSCS can be a promising rehabilitative tool for people with SCI.

The commercially available electrodes employed for tSCS, however, are relatively large resulting in a wider distribution of the applied current. Additionally, being placed over the skin, the electrodes are also distant from the dorsal fibers of the spinal cord with layers of intervening connective and bony tissue including the dorsal aspects of the cervical vertebrae. These factors may limit the effectiveness and specificity of tSCS for the recruitment of target motoneuron pools which could be a major drawback since epidural stimulation studies have shown the importance of targeted stimulation (Wagner et al., 2018; Rowald et al., 2022) in restoring voluntary muscle control. Recent efforts focusing on appropriate placement of these relatively large tSCS electrodes have shown some effectiveness in selective recruitment of the dorsal spinal roots (Oh et al., 2022; Bryson et al., 2023).

We performed targeted transcutaneous stimulation of the cervical spinal cord paired with weekly activity-based training in two individuals with motor-complete SCI. We used a custom electronically-configurable electrode array, with each electrode having a small form factor (10 mm x 10 mm). To target individual cervical levels, stimulation was provided simultaneously through 3 adjacent electrodes within a single row, spanning the midline. This spatial configuration enabled the characterization of the recruitment profile of the upper-limb motor pools along the rostrocaudal axis. The stimulation location to achieve maximal recruitment of the muscle group of interest was chosen based on this recruitment profile for subsequent activity-based training. Participants performed motor training involving isometric contractions of the target muscles while receiving tSCS once per week. Even with a once-per-week regimen, we observed a rapid increase in both volitionally controlled muscle activity, up to a 1,136% increase in effective force and 2-point increase in somatosensation within a period of 5–6 weeks. However, the observed gains were restricted to muscles that showed a discernable force production during the pre-intervention assessment.

Taken together, this study demonstrates the advantages of tSCS using a highly configurable electrode array in conjunction with a weekly activity-based training regimen in restoring volitional control of upper-limb movement and sensation in people with SCI.

## Methods

### Participants

We performed tSCS of the cervical spinal cord in two individuals with tetraplegia resulting from C5 level motor-complete SCI. The details of the participants are summarized in Table 1. Their clinical assessment scores taken pre-intervention, at the end of intervention and at follow-up sessions are provided in Supplementary Tables 1, 2, respectively. All procedures were approved by the Northwell Health Institutional Review Board. The study was conducted in accordance with the principles embodied in the Declaration of Helsinki and in accordance with local statutory requirements. All participants provided formal written informed consent to participate in this study. The study has been registered with ClinicalTrials.gov (NCT04755699, first posted on 16/02/2021).

**Table 1:** Study participants information.

Participant	Age range	Gender	Injury type	Time since injury (yrs.)
CTS02	early 20s	M	SCI: C5, ASIA A	4
CTS03	mid 30s	M	SCI: C5, ASIA B	7

Demographic and injury-related information for each participant.

A complete ISNCSCI assessment had been performed for both participants in the clinic as part of their regular course of treatment. We used this assessment as part of the eligibility assessment of each participant. We did not monitor the ISNCSCI scores for levels below T1 once the intervention started. Both participants had motor and sensory impairments at a neurological level of C5 on both sides. For CTS02, the motor and sensory zones of partial preservation were C8 and at least T1 on both sides, respectively. For CTS03, the motor and sensory zones of partial preservation were C7 and T1 on both sides, respectively. We did not explicitly explore where the zones of partial preservation extended beyond T1 as stated above.

### Intervention protocol

#### Before intervention period

Baseline clinical assessment, namely the Graded Redefined Assessment of Strength, Sensation and Prehension (GRASSP), was performed before the onset of any intervention. These assessments were repeated for two sessions, scheduled a month apart, to establish a pre-intervention baseline.

#### Start of the intervention period

At the beginning of the intervention period, we characterized the tSCS-mediated muscle recruitment profile for each participant. tSCS consisted of a 10 kHz biphasic sinusoidal waveform with a pulse duration of 1 ms. Low frequency stimulation (3 Hz) of increasing current amplitudes was sequentially targeted at different locations along the rostrocaudal axis of the cervical region and the resultant compound action potentials from specific muscles of the arm and hand were recorded. Based on the specificity of tSCS in recruiting the different motoneuron pools, we choose the specific location where we subsequently localized the tSCS to be paired with the activity-based training.

#### Intervention period

After the pre-intervention clinical assessment baselines were established, we began the intervention period wherein study participants attended 4-h long experimental sessions in the lab once per week. To evaluate the therapeutic effects of tSCS, we focused on the triceps brachii for each of our study participants. At the beginning of every session, we measured both the evoked force and electromyographic (EMG) activity in these target muscles before administering any spinal cord stimulation. Following that, participants received transcutaneous spinal cord stimulation (tSCS) in the cervical region for 1 h each session. During tSCS, participants were administered activity-based training wherein they performed isometric contractions specifically designed to activate the target muscles. The intervention period in this manuscript took place across 35 weeks.

#### ‘No Stim’ period

After 16 weeks of receiving of continuous tSCS, we administered a 3-week period when the participant received no stimulation. They continued to perform the tasks that constituted their activity-based training. Weekly administration of tSCS was resumed after this ‘No Stim’ period.

#### End of intervention period

tSCS was resumed after the *No Stim* period up to the end of 35 weeks since the beginning of intervention. Force and EMG was recorded as before.

### EMG signal processing

Bipolar surface EMG electrodes were used to record muscle activity from the following muscles of the left arm and hand: biceps brachii (BIC), triceps brachii (TRI), flexor digitorum superficialis (FDS), extensor digitorum communis (EDC) and abductor policis brevis (APB). We chose these muscles as they are primarily innervated by the cervical roots, namely BIC: C5, TRI: C7, FDS: C8, EDC: C7, ABP: C7. We used pre-gelled Ag/AgCl electrodes (Ø 24 mm, Arbo™ H124SG, Covidien), connected to EMG sensors (AT-04-001 MyoWare™ Muscle Sensor), a differential amplifier (AD6221) with a 2nd order bandpass filter (20-498 Hz), and a signal digitizer (PicoScope® Model 4824A). The MyoWare sensor required the electrodes to have a center-to-center distance of 30 mm. The sampling rate used was 10 MHz while characterizing the recruitment profile of the upper-limb motoneuron pools and 10–20 kHz during activity-based training. For characterizing the area-under-the curve (AUC), the EMG signal was digitally filtered using a 60 Hz infinite impulse response (IIR) comb filter and a 6^th^-order Butterworth bandpass filter between 10 and 1,000 Hz in MATLAB.

### Transcutaneous spinal cord stimulation (tSCS)

**Figure 1:**
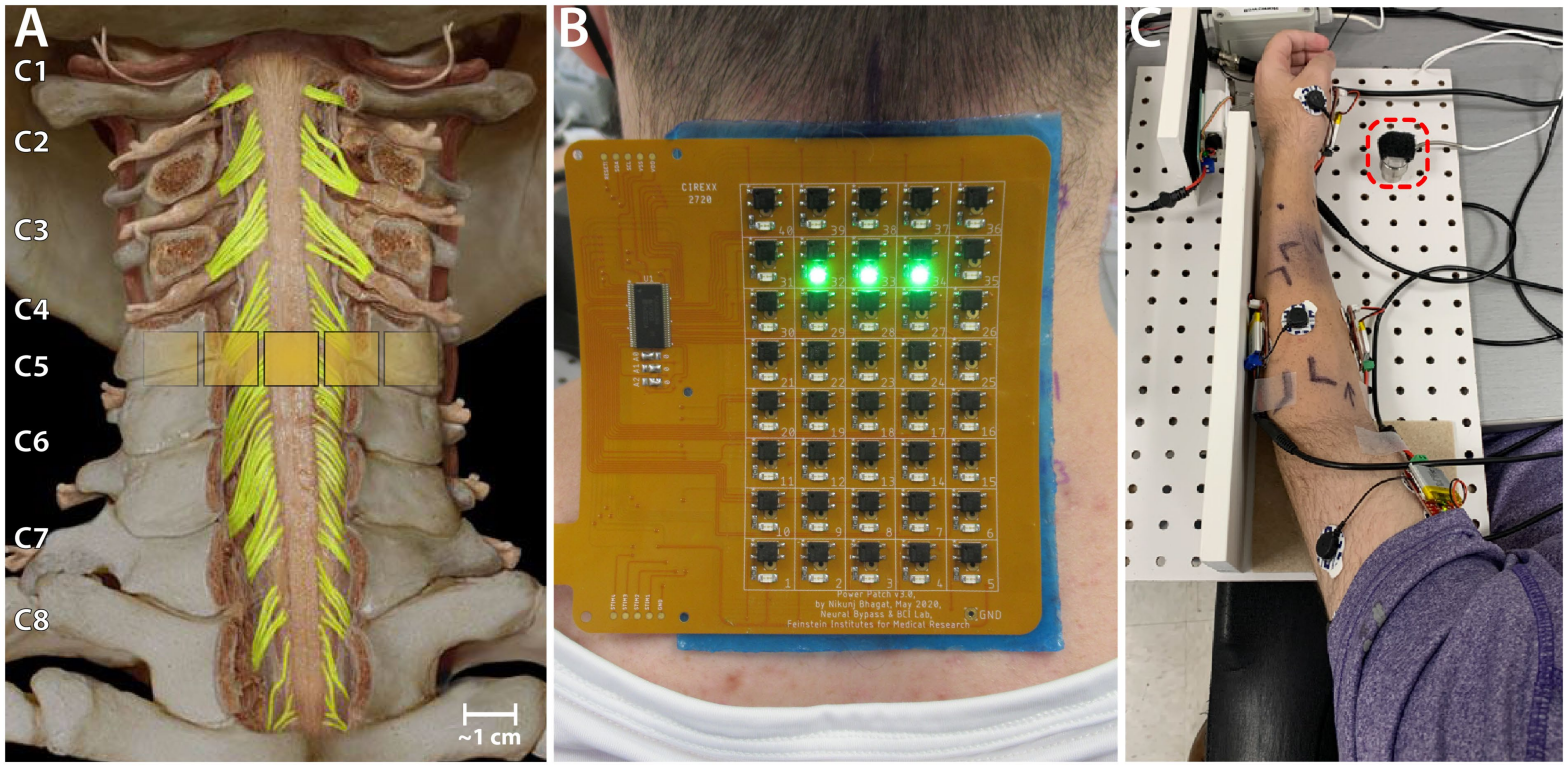
Experiment setup. **(A)** Schematic showing the location of a 1 × 3 activated electrode configuration superimposed over the human spinal cord showing the dorsal column and roots. **(B)** The custom electronically configurable electrode array placed over the cervical spinal cord of a study participant with a 1 × 3 configuration of activated electrodes (green LEDs). **(C)** Setup for measuring triceps force. The ulnar protrusion of the wrist is placed over the 25 lb. load cell during the task (red dashed square).

Transcutaneous spinal cord stimulation was provided using a custom stimulator and electrode array. The stimulator consisted of a microcontroller to produce the stimulation waveform digitally, a Texas Instruments class-D amplifier (TAS5825P) in voltage mode, and a 12:1 step-up transformer (Xicon 42TM003-RC). The stimulator was powered by a 24 V battery and a safety circuitry was used to restrict power density levels to no more than 0.25 W/cm^2^. The flexible PCB electrode array consisted of electroless nickel immersion gold (ENIG) or immersion silver-plated square contacts (10 mm x 10 mm) arranged in an 8 × 5 pattern with a 11 mm center-to-center electrode separation. Electrical stimulation to any combination of the 40 electrodes within the array, was controlled using solid-state relays mounted above each of the electrodes and a custom MATLAB-based GUI. Miniature green LEDs mounted on the dorsal surface of the electrode array, visually indicated the active electrodes. To target specific cervical segments (Figure 1A), an electrode configuration of 1 × 3 (Figure 1B) was used wherein stimulation was provided simultaneously to 3 adjacent electrodes within a single row, spanning the midline. The electrode array was affixed to the back of the neck using a rectangular piece of proprietary hydrogel. To ensure consistency in placement of the array between sessions, we used the inion of the external occipital protuberance as a landmark. Distances measured from the inion were used to place the electrode array and identify the location of stimulation. Two interconnected 5 × 10 cm rectangular self-adhesive hydrogel electrodes (Axelgaard Manufacturing Co., Ltd., USA) placed along the midline over the lumbar spinal cord served as return electrodes (anodes).

Stimulation consisted of a 10 kHz biphasic sinusoidal waveform with pulse duration of 1 ms delivered at 3 Hz for generating recruitment profiles.

Stimulation frequency was set to 50 Hz when tSCS was paired with activity-based training and the pulse width was reduced to 0.5 ms to reduce neck muscle activation and increase participant comfort. Stimulation amplitude was gradually raised up to the maximal level that the participants found tolerable while not interfering with their training which was usually 140–160 mA. The desired level of current was reached by manually adjusting the voltage output of the stimulator while continuously monitoring the current output on an electrically-isolated oscilloscope. tSCS paired with activity-based training was administered for 45–60 min per session.

### Experimental sessions

Heart rate and blood pressure were measured at the beginning and end of each session. For finer measurements of voluntary muscle control, we measured both the evoked force and electromyographic (EMG) activity in the muscles of the hand and arm. For efficiency and study homogeneity alone, we uniformly chose to monitor the left arm for both participants and configured our system accordingly. The tasks performed as a part of their activity-based training involved generating maximal force using their triceps brachii during isometric elbow extension. The participants’ arms were extended out in front of them, with the elbow flexed at 30°as this was the most reliable position to measure isometric triceps brachii muscle strength (Prkić et al., 2018). The hand was held in a neutral position and a load cell (load range of 25 lb., Model M31, Honeywell International, Inc.) placed under the ulnar head prominence at the wrist (Figure 1C). The participants were instructed to push down on to the load cell in this position. The elbow was resting on the table and prevented from lifting during the task.

For all force measuring tasks, the experimenter cued the participants to push against the force sensor for 3–5 s and then to relax for 3–5 s. The task was performed in 2 sets of 5 trials each with a 60–90 s of rest period between the sets. Verbal encouragement was provided to encourage the participants to generate maximal sustainable force. The sensors used were calibrated at regular intervals using standard weights (Supplementary Figure 1).

While receiving tSCS, the participant performed the same tasks as described above. This served the dual purpose of providing activity-based training while receiving tSCS as well as measuring the volitional EMG activity and force.

### tSCS-mediated muscle recruitment profile

Stimulation for generating recruitment profiles was delivered at 3 Hz with a pulse duration of 1 ms (the resultant duty cycle of 0.3% generates an average power density level that is well below the safety limits of 0.25 W/cm^2^).

Stimulation amplitudes tested ranged from 100 mA to up to 225 mA in intervals of approximately 25 mA. This was repeated for each of the eight rows of electrodes on the array. The Picoscope 6 acquisition software (Pico Technology, Cambridgeshire, UK) was used to trigger acquisition of a 100 ms-long EMG signal following each stimulation pulse. For each stimulation amplitude and at each electrode row, we recorded such an EMG signal from 20 to 30 repetitions of the stimulation pulse. All the data was imported into MATLAB for further analysis. From each of the EMG signals recorded, we isolated a snippet starting from 5 ms and ending at 55 ms after the stimulation artifact. We measured the peak-to-peak amplitude (P2P) for this snippet. It was included for analysis only if the maximum amplitude of the snippet was greater than 5 times the standard deviation of baseline signal of that recording channel. For each muscle, the P2P amplitudes were normalized to the maximal P2P amplitude recorded across all amplitudes and electrode rows.

X-ray images for both participants in the sagittal plane with radio-opaque markers on the neck were obtained to determine actual location of electrodes with respect to spinal roots and vertebral landmarks.

We also measured the stimulation current of maximal activation for each muscle. For this, at each cervical level, we increased the amplitude up to 300 mA until the reflexive activation of the muscle was visually determined to be consistent for every stimulation pulse and did not change for any small increase in amplitude. This stimulation amplitude was noted as the Maximal Activation Current for that muscle at the cervical level. This procedure was repeated for each muscle.

### Functional outcome

GRASSP strength and sensibility assessments were performed every 4 weeks. The prehension assessment subtest was performed only at the beginning of the study to ascertain baseline capabilities. The prehension assessment subtest was not included in the subsequent assessments as there was no overt recovery in finger movement or hand function in either participant during the course of the study. In addition to the areas recommended in the GRASSP assessment for sensibility evaluation, we tested a few additional relevant spots on the volar aspect of the hand to obtain a comprehensive profiling of the sensory changes in the entire hand. Specifically, we included the remaining finger pads and four corners of the palm.

For determining the strength and sensibility scores at the end of administering tSCS, we averaged the GRASSP scores from the last 3 assessments performed while the participants received tSCS. These assessments spanned a period of up to 2 months before the end of intervention. We also performed up to 2 follow-up GRASSP assessments post-termination of administering tSCS. These scores were compared against the baseline scores.

### Statistical analysis

For statistical analysis of the evoked force, we compared the forces recorded in three stages of the study (start, no-stim and end of study). For the early stage, we chose the first three consecutive sessions after start of weekly tSCS sessions. For the no-stim stage, we chose three sessions during the period when tSCS was not being delivered. For the end of study stage, we chose the last three consecutive sessions. We performed a repeated-measures Analysis of Variance (ANOVA) between the average values of force obtained in the three epochs. We employed the Bonferroni correction for all multiple comparisons. As the presented work is pilot in nature, statistical analysis is limited.

## Results

Our results demonstrate that transcutaneous spinal cord stimulation can be used to activate specific motoneuron pools in the cervical spinal cord. Moreover, targeted tSCS paired with minimal activity-based training resulted in a substantial and sustained increase in muscle activity and strength in specific upper-limb muscles in two patients with motor-complete cervical SCI.

### Recruitment of upper limb muscles through targeted tSCS

**Figure 2:**
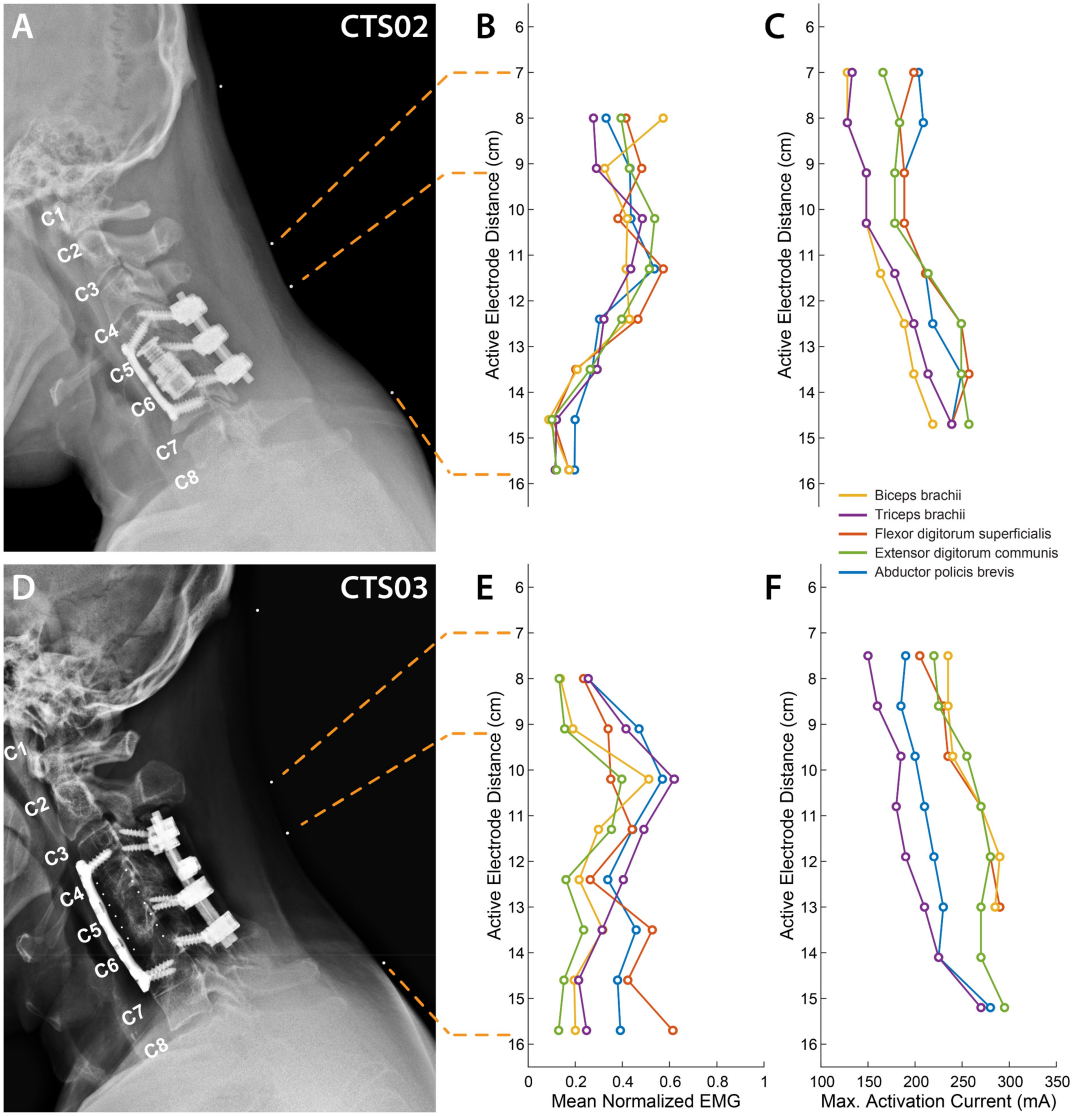
Muscle recruitment profile during cervical tSCS. **(A)** X-ray images for participant CTS02 in the sagittal plane with radio-opaque markers on the neck (white dots). The topmost marker identifies the inion of the external occipital protuberance. The second and third markers identify points 7 cm and 9.2 cm respectively, from the inion signifying the first and third rows of a putative electrode array whose first row of electrodes was aligned at 7 cm from the inion. The last marker identifies the location of the last row of the electrode array at 15.7 cm from the inion. The cervical labels mark the exit point of the respective dorsal roots. **(B)** Mean activation of the 5 muscles across all stimulation amplitudes mediated by tSCS through each of the 8 electrode rows for CTS02. **(C)** Stimulation amplitude that resulted in maximal activation of each of the 5 muscles for CTS02. **(D)** X-ray images for participant CTS03. **(E)** Mean activation of the 5 muscles across all stimulation amplitudes mediated by tSCS through each of the 8 electrode rows for CTS03. **(F)** Stimulation amplitude that resulted in maximal activation of each of the 5 muscles for CTS03.

We characterized the recruitment profile of the motoneuron pools innervating the upper-limb muscles. To determine the effect of the location of stimulation along the rostro-caudal axis on the recruitment of the upper-limb muscles, we sequentially delivered stimulation of varying amplitudes to different electrode triplets in the array. An X-ray image with radio-opaque markers on the neck (Figures 2A,D) shows a subset of the stimulation locations. Simultaneously, we recorded EMG activity from 5 muscles of the left arm and hand (see Supplementary Figure 2 for an example tSCS-evoked EMG activity.). We hypothesized that the recruitment pattern of the upper-limb muscles through transcutaneous stimulation would reflect the rostrocaudal segment-wise distribution of the upper-limb motor nuclei in the cervical spinal cord. The recruitment profiles showed a distinct and consistent shape in both participants. Almost all the muscles exhibited the best recruitment when stimulation was delivered around the C5-C6 level. Stimulating using rostral-most electrodes located at the C4-C5 level primarily activated the biceps in CTS02, while in CTS03 the biceps were activated the strongest by stimulating the C5-C6 level. In both participants, activation of the triceps brachii was strongest when stimulation was localized around the C5-C6 level. Stimulation using electrodes positioned more caudally required higher currents but resulted in decreased recruitment across all muscles. Recruitment of FDS and APB occurred with stimulation at C7-C8 level stimulation in participant CTS02 (Figures 2B,C), and C8-T1 level in participant CTS03 (Figures 2E,F). The fact that the participants had a spinal cord injury and metallic implants in the cervical vertebrae did not result in an obvious difference in the recruitment profile of the upper-limb motor pools is noteworthy. Additionally, we also determined the current required for maximal activation (Max. Activation Current) for each of the upper limb muscles. The thresholds of activation showed similar results as the recruitment profiles with biceps and/or triceps brachii having a lower threshold of activation during rostral stimulation (Figures 2C,F). The participants did not report any acute discomfort or lasting effects from the current levels in this experiment.

### Increased muscle activity and force generated with tSCS

Since previous spinal cord stimulation studies have generally shown improvements in muscles that have at least some residual activity, we focused on the volitional control of the triceps muscle in our participants. For both participants, we delivered tSCS targeted at the C6 level (about 10 cm from the inion of the external occipital protuberance) as stimulation at this level showed maximal recruitment of the triceps muscle.

**Figure 3:**
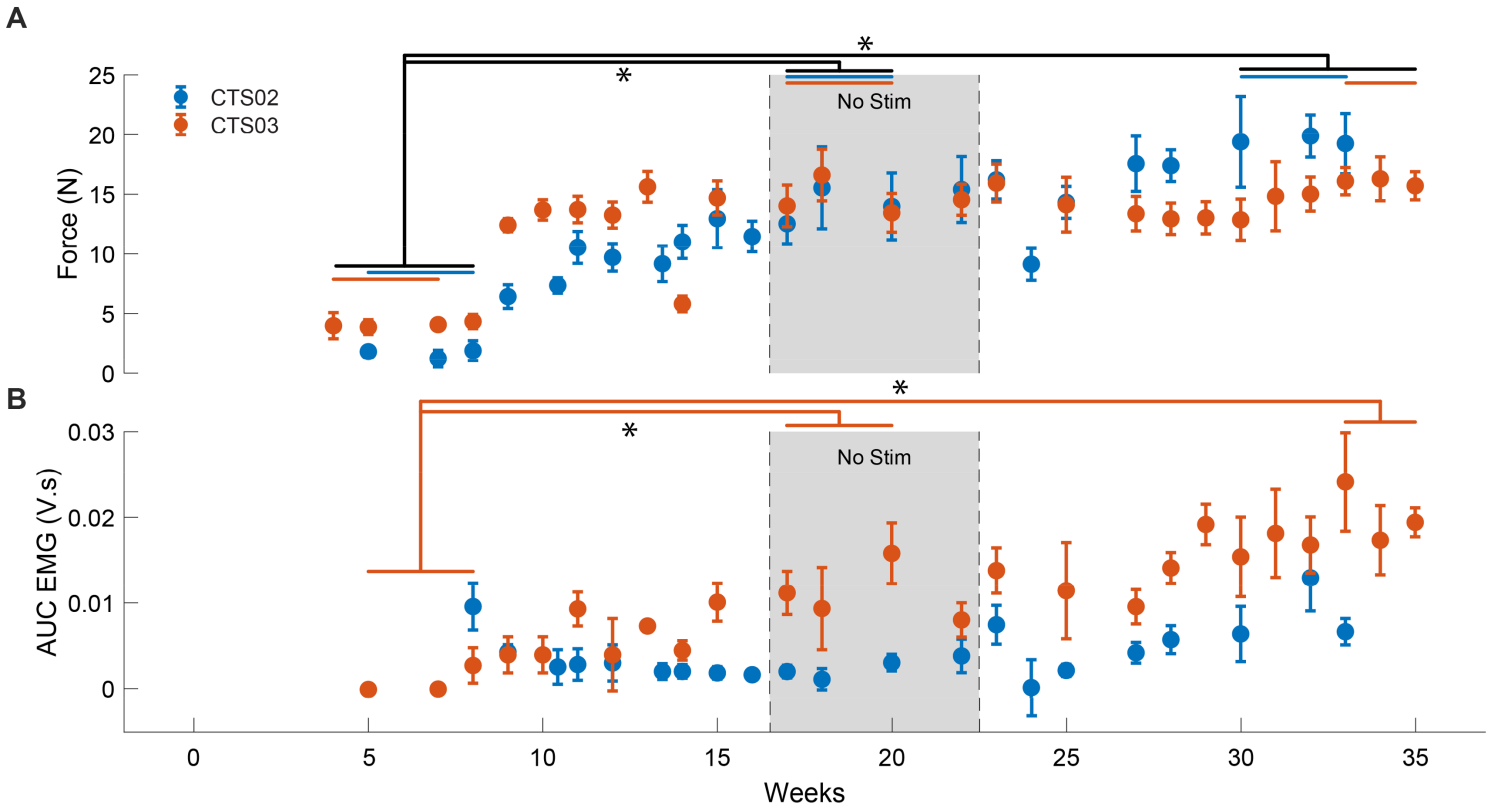
Increase in muscle activity and force after tSCS. **(A)** The force generated by the left triceps muscle in case of participant CTS02 (blue) and CTS03 (orange). * indicates significant difference (Repeated measures ANOVA and Bonferroni correction for multiple comparison). The blue (CTS02) and orange (CTS03) bars indicate the specific data points from each participant that were included for the statistical analysis. **(B)** The AUC of the EMG activity recorded from the left triceps muscle during isometric triceps flexion. * indicates significant difference (t-test with Bonferroni correction for multiple comparisons).

We observed an average increase of force generated by the left triceps muscle of up to 893% (at the end of 17 sessions) and 825% (at the end of 16 sessions) for CTS02 and CTS03, respectively, in the ‘No Stim’ period compared to the start of the study. This increased to 1,136% (29 sessions) and 1,035% (31 sessions), respectively (*F* = 13.99, *p* < 0.01), (Figure 3A; Supplementary Figure 3). We also observed a corresponding increase in EMG activity for the triceps muscle (*F* = 11.035, *p* < 0.01) (Figure 3B). But, this was primarily driven by the increase in EMG activity observed in CTS03 (t-statistic = −8.76, *p* < 0.01 between ‘No Stim’ period and start of study; t-statistic = −8.78, *p* < 0.01 between the end of study and start of study).

### GRASSP assessments

**Figure 4:**
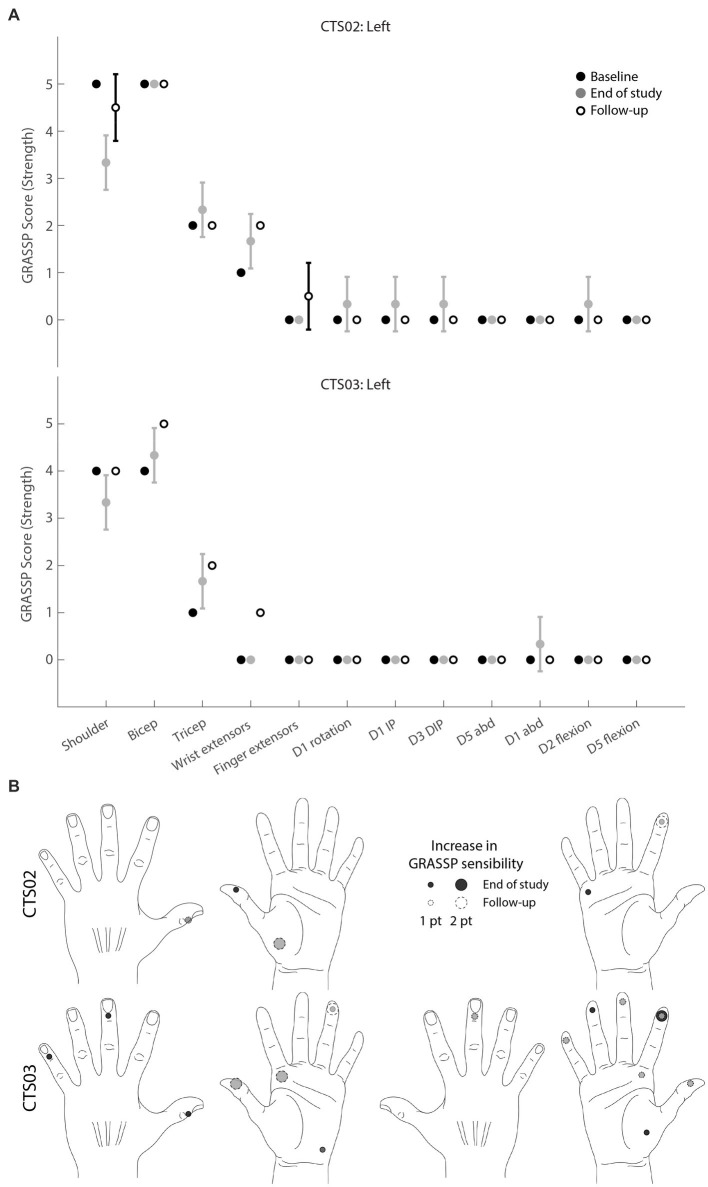
GRASSP assessments. **(A)** GRASSP strength change from baseline. Black circles indicate mean baseline strength score (*n* = 2). Gray circles indicate mean strength score at the end of study (*n* = 3), White circles indicate mean monthly follow-up assessments (*n* = 2 for CTS02; *n* = 1 for CTS03). Error bars indicate standard deviation **(B)** GRASSP sensibility change from baseline scores (*n* = 2). Circles show 1- to 2-point increase in sensory perception as defined by the GRASSP sensibility scale. Black circles indicate change at end of study (*n* = 3); white circles with dashed lines indicate change observed at follow-up assessments (*n* = 2). Gray shows overlap.

To evaluate the clinical significance of the progress showed by each participant, we also performed standard clinical assessments, namely the GRASSP test. The GRASSP strength test showed modest increases in the motor functions assessed, including the triceps muscle which was specifically targeted in this study (Figure 4A). The trace movements observed in the fingers were not replicated in the follow-up assessments. However, some of the gains in the upper arm muscles, specifically wrist extensors in CTS02 and triceps in CTS03, persisted even during the follow-up assessments. The GRASSP sensibility test showed up to a 2-point increase in sensitivity scores in the palmar regions of the hand in both participants at the end of the intervention. The follow-up assessments found many of these gains to have persisted even after the termination of the intervention (Figure 4B). Participants also shared their anecdotal descriptions of the effect of receiving tSCS. They described increased control in moving their arms and being able to give “stronger hugs.”

## Discussion

In this study after delivering tSCS, we observed a substantial increase in volitionally generated force, and significant increases in tactile sensation in two individuals with SCI. Both participants showed these changes within a 6-8-week period of receiving tSCS. Interestingly, the improvements were restricted to the muscles that showed discernable force production during the pre-intervention assessment. We did not observe any improvement in the forces and EMG activity generated during finger flexion in either participant with SCI even when the activity was performed over many weeks while receiving tSCS. This could be due to the severity of the injuries being too great to benefit from this type of therapeutic intervention (Huang et al., 2022).

Our results are comparable to those demonstrated by other studies involving cervical tSCS in people with SCI (Freyvert et al., 2018; Inanici et al., 2021; Huang et al., 2022). However, our design involved participants receiving spinal cord stimulation and activity-based training for 1–2 h only, once every week. Importantly, our stimulation was targeted to achieve maximal recruitment of the muscle group of interest. This suggests that targeted tSCS could improve the efficacy of spinal cord activation and achieve restoration of volitional control even with a weekly activity-based training regimen.

The electrode configuration we used in this study was a 1 row x 3 columns configuration spanning the spinal cord midline. The effectiveness of such an electrode configuration in recruitment of specific motor neuron pools has been demonstrated in able-bodied individuals (Gerasimenko et al., 2015; Krenn et al., 2015). It is highly likely that this configuration resulted in the stimulation of the dorsal column fibers thereby activating motor nuclei in off-target cervical levels and thus reducing specificity. Activation of the dorsal roots via lateralized stimulation could result in greater selectivity in the motoneuron pools being activated (Oh et al., 2022; Bryson et al., 2023). However, an earlier study with lateralized stimulation showed an increase in side-specific activation and not across spinal levels (Calvert et al., 2019). Previous studies have explored the idea of targeting stimulation above and below the injury level with the aim of enhancing the activity of the descending inputs as well as local circuitry below the site of injury (Zhang et al., 2020; Inanici et al., 2021). With a high cervical location of SCI (C5) for the participants in this study, most of the stimulation was restricted to being targeted at or below the injury level (Freyvert et al., 2018). In fact, stimulation targeted at higher cervical levels was perceived as uncomfortable by the participants. This demonstrates that tSCS can be tailored to suit patient comfort, target only those cervical levels innervating the muscle of interest, and still result in significant motor improvements.

Furthermore, we developed and utilized a custom electrode array that could be electronically configured. This allowed efficient mapping and can support dynamic spatial pattern switching. In future studies, this feature can be combined with brain-computer interfaces (BCIs) to switch spatial patterns based on movement-related information decoded from intracortical activity (Bouton et al., 2016). Such automated configuration of targeted tSCS based on user movement intentions would further optimize the stimulation pattern and could improve the therapeutic outcomes, along with the usability of this technology by people with SCI while performing activities of daily living at home.

The 3-week ‘No Stim’ period allowed us to evaluate whether the gains in muscle strength would persist after withdrawing weekly stimulation. Increasing this period would be of interest in future studies. The statistically significant improvement in the force generated suggests that even 16 weeks of receiving tSCS can evoke persistent benefits.

In this study, we could not rule out that lower motoneuron damage precluded any kind of motor improvement for the finger flexor muscles in case of participants with SCI. A nerve-conduction measurement should always accompany such studies to characterize the state of the lower motoneurons before, during and after the study. We regularly confirmed with the participants that there were no major changes to their regular schedule of physical therapy or activities of daily living. However, it is still possible the motivational effects of being involved in a study by itself could have boosted participants involvement in their regular physical therapy. However, it is not always possible to have a control arm for such studies. Such a drawback needs to be clearly recognized while interpreting these results.

As much as the participants gained in volitionally generated force, the GRASSP scores corresponding to the triceps showed only a modest increase. We venture that the low resolution of the GRASSP scores and the strict criteria that is required to move up the GRASSP scale is the reason for this discrepancy. The participants also showed some variability in their GRASSP scores from one assessment session to the next, fluctuating between two adjacent GRASSP strength levels. This was probably the reason for the surprising drop in shoulder strength score and does not reflect an actual loss in shoulder strength as is borne out by the follow-up assessments.

It was interesting to observe improvement in tactile sensation for regions innervated by spinal roots from below the injury level in both participants with SCI. To our knowledge, this is the first study to document improvements in tactile sensation as measured by the GRASSP sensibility test in people with SCI after receiving tSCS. Stimulating dorsal roots of the spinal cord has been demonstrated to relay somatotopically relevant sensory information (Chandrasekaran et al., 2020). Meanwhile, restoring somatosensation through intracortical stimulation has been demonstrated in humans as well (Flesher et al., 2016; Chandrasekaran et al., 2021; Fifer et al., 2022). It may be worthwhile to explore the benefits of pairing intracortical stimulation and tSCS for the long-term rehabilitation of somatosensation in SCI.

## Data availability statement

The raw data supporting the conclusions of this article will be made available by the authors, without undue reservation.

## Ethics statement

The studies involving human participants were reviewed and approved by Northwell Health Institutional Review Board. The patients/participants provided their written informed consent to participate in this study.

## Author contributions

SC, NB, and CB designed the study. SC, NB, RR, SE, and CB performed the experiments. SC analyzed the data. NB designed and tested the electrode array, with guidance from CB. DG performed independent assessments of the motor and sensory capacity of the participants. PS and SH provided critical feedback and input. SC and CB finished the initial draft of the manuscript. All authors contributed toward interpreting the results of the experiments, provided critical review, edits, and approval of the final manuscript.

## Funding

This study was funded through support provided by Feinstein Institutes for Medical Research at Northwell Health.

## Conflict of interest

CB has multiple patents in related fields and financial interests and/or involvement in multiple start-up companies including Sanguistat, myString, and Neuvotion. SE has financial interests in Neuvotion, Inc.

The remaining authors declare that the research was conducted in the absence of any commercial or financial relationships that could be construed as a potential conflict of interest.

## Publisher’s note

All claims expressed in this article are solely those of the authors and do not necessarily represent those of their affiliated organizations, or those of the publisher, the editors and the reviewers. Any product that may be evaluated in this article, or claim that may be made by its manufacturer, is not guaranteed or endorsed by the publisher.
