# Supplementary material for: Targeted transcutaneous spinal cord stimulation promotes persistent recovery of upper limb strength and tactile sensation in spinal cord injury: a pilot study
Source: Front Neurosci. 2023 Jul 7;17:1210328. doi: 10.3389/fnins.2023.1210328 (PMC10360050; doi:10.3389/fnins.2023.1210328)
Supplement: Supplementary file 1 [file Table_1.pdf]

GRASSP Movement	Pre-Intervention Baseline 1		Pre-Intervention Baseline 2		At End of Intervention		1-month follow-up		2-month follow-up	
	**Left**	**Right**	**Left**	**Right**	**Left**	**Right**	**Left**	**Right**	**Left**	**Right**
**Shoulder**	5	5	5		4	4	4	5	5	5
**Biceps**	5	5	5		5	5	5	5	5	5
**Triceps**	2	2	2		2	1	2	2	2	2
**Wrist extensors**	1	1	1		2	2	2	1	2	1
**Finger extensors**	0	0	0		0	0	0	1	1	0
**D1 rotation**	0	0	0		0	0	0	0	0	1
**D1 IP**	0	0	0		0	0	0	0	0	0
**D3 DIP**	0	0	0		0	0	0	0	0	0
**D5 abduction**	0	0	0		0	0	0	0	0	0
**D1 abduction**	0	0	0		0	1	0	1	0	0
**D2 flexion**	0	0	0		0	0	0	0	0	0
**D5 flexion**	0	0	0		0	0	0	0	0	0
**GRASSP Sensation**										
	**Left**	**Right**	**Left**	**Right**	**Left**	**Right**	**Left**	**Right**	**Left**	**Right**
1 (D1 dorsum)	3	3	2	3	4	3	3	3	4	4
2 (D3 dorsum)	0	0	0	0	0	0	1	0	1	0
3 (D5 dorsum)	2	0	3	0	0	0	1	0	2	0
**Dorsal Total (12)**	**5**	**3**	**5**	**3**	**4**	**3**	**5**	**3**	**7**	**4**
4 (D1 palmar)	3	3	3	3	4	3	3	3	3	2
5 (D3 palmar)	0	1	0	3	1	0	0	1	0	1
6 (D5 palmar)	0	0	1	0	0	0	1	0	0	0
**Palmar Total (12)**	**3**	**4**	**4**	**6**	**5**	**3**	**4**	**4**	**3**	**3**
**Total (out of 24)**	**8**	**7**	**9**	**9**	**9**	**6**	**9**	**7**	**10**	**7**
Index	4	0	2	0	1	2	2	1	3	2
Ring	1	2	1	0	0	3	0	0	1	0
Palm (Index)	3	0	0	1	1	0	0	1	0	1
Palm (Pinky)	3	0	0	0	1	0	0	0	0	0
Palm (Base)	4	3	0	0	1	2	1	1	3	2
Palm (Thumb)	0	3	1	3	4	3	1	1	4	2

Supplementary Table 1. GRASSP movement and sensation scores for CTS02
