# Supplementary material for: Targeted transcutaneous spinal cord stimulation promotes persistent recovery of upper limb strength and tactile sensation in spinal cord injury: a pilot study
Source: Front Neurosci. 2023 Jul 7;17:1210328. doi: 10.3389/fnins.2023.1210328 (PMC10360050; doi:10.3389/fnins.2023.1210328)
Supplement: Supplementary file 2 [file Table_2.pdf]

GRASSP movement	Pre-Intervention Baseline 1		Pre-Intervention Baseline 2		At End of Intervention		1-month follow-up		2-month follow-up	
	**Left**	**Right**	**Left**	**Right**	**Left**	**Right**	**Left**	**Right**	**Left**	**Right**
**Shoulder**	4	2	4		3	1	4	2		
**Biceps**	4	2	4		4	2	5	3		
**Triceps**	1	2	1		2	2	2	2		
**Wrist extensors**	0	0	0		0	1	1	0		
**Finger extensors**	0	0	0		0	1	0	0		
**D1 rotation**	0	0	0		0	0	0	0		
**D1 IP**	0	0	0		0	0	0	0		
**D3 DIP**	0	0	0		0	0	0	0		
**D5 abduction**	0	0	0		0	0	0	0		
**D1 abduction**	0	0	0		1	0	0	1		
**D2 flexion**	0	0	0		0	0	0	0		
**D5 flexion**	0	0	0		0	0	0	0		
**GRASSP Sensation**										
	**Left**	**Right**	**Left**	**Right**	**Left**	**Right**	**Left**	**Right**	**Left**	**Right**
1 (D1 dorsum)	3	4	4	4	4	4	3	3	4	4
2 (D3 dorsum)	3	3	4	2	4	4	4	3	2	4
3 (D5 dorsum)	2	4	4	4	4	4	2	4	1	4
**Dorsal Total (12)**	**8**	**11**	**12**	**10**	**12**	**12**	**9**	**12**	**7**	**12**
4 (D1 palmar)	1	3	1	4	3	4	4	4	2	4
5 (D3 palmar)	2	3	2	3	2	4	1	4	2	4
6 (D5 palmar)	2	4	3	3	3	4	2	4	2	4
**Palmar Total (12)**	**5**	**10**	**6**	**10**	**8**	**12**	**7**	**12**	**6**	**12**
**Total (out of 24)**	**13**	**21**	**18**	**20**	**20**	**24**	**16**	**22**	**13**	**24**
Index	2	3	1	2	2	4	2	3	2	4
Ring	2	4	2	3	4	4	4	3	4	3
Palm (Index)	0	4	0	2	2	4	2	3	2	4
Palm (Pinky)	3	4	3	4	3	4	3	3	4	3
Palm (Base)	3	4	2	4	4	4	2	3	2	3
Palm (Thumb)	3	3	3	4	3	4	4	3	3	4

Supplementary Table 2. GRASSP movement and sensation scores for CTS03
