# Supplementary material for: Targeted transcutaneous spinal cord stimulation promotes persistent recovery of upper limb strength and tactile sensation in spinal cord injury: a pilot study
Source: Front Neurosci. 2023 Jul 7;17:1210328. doi: 10.3389/fnins.2023.1210328 (PMC10360050; doi:10.3389/fnins.2023.1210328)
Supplement: Supplementary file 3 [file Image_1.pdf]

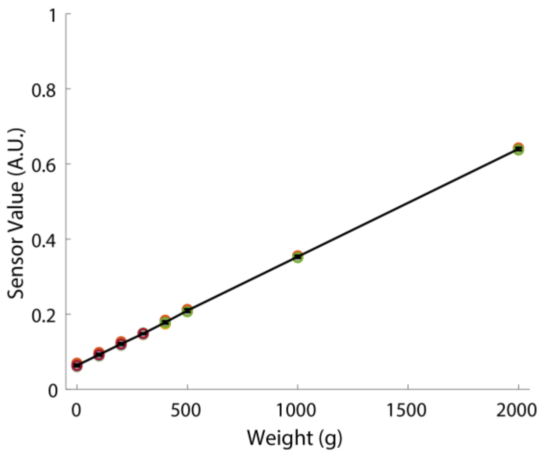

Supplementary Figure 1. Calibration curves for the sensor used in this study. Each color signifies a calibration test done on a different day. Black line signifies average calibration across days. Error bars shown are standard deviations.
