# Supplementary material for: Targeted transcutaneous spinal cord stimulation promotes persistent recovery of upper limb strength and tactile sensation in spinal cord injury: a pilot study
Source: Front Neurosci. 2023 Jul 7;17:1210328. doi: 10.3389/fnins.2023.1210328 (PMC10360050; doi:10.3389/fnins.2023.1210328)
Supplement: Supplementary file 4 [file Image_2.pdf]

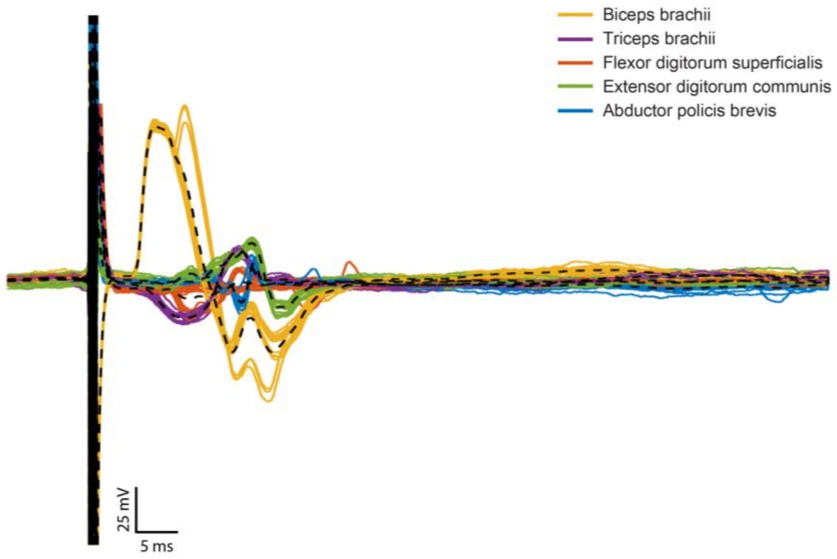

Supplementary Figure 2. Example trace of muscle recruitment profile of the 5 muscles upon tSCS stimulation. This was for participant CTS02 when stimulated at 10.2 cm at 150 mA. Colored traces indicate the reflex activity evoked in each of the 5 muscles as shown in the legend. Dashed black line shows average of all the trials.
