# Supplementary material for: Targeted transcutaneous spinal cord stimulation promotes persistent recovery of upper limb strength and tactile sensation in spinal cord injury: a pilot study
Source: Front Neurosci. 2023 Jul 7;17:1210328. doi: 10.3389/fnins.2023.1210328 (PMC10360050; doi:10.3389/fnins.2023.1210328)
Supplement: Supplementary file 5 [file Image_3.pdf]

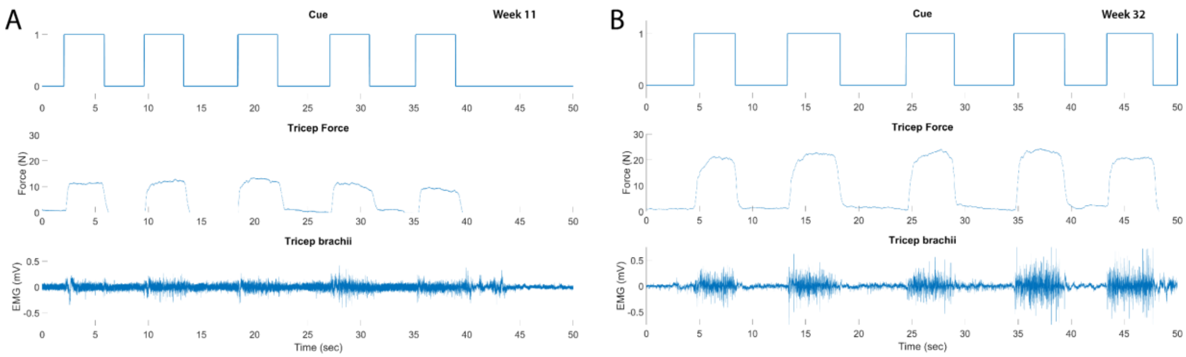

Supplementary Figure 3. Example traces of cue, force measured during tricep extension and EMG recorded from triceps brachii for participant CTS02 from A) week 11 and B) week 32.
